# Optimization of Bangladesh and Malaysian genotype recombinant reporter Nipah viruses for *in vitro* antiviral screening and *in vivo* disease modeling

**DOI:** 10.1016/j.antiviral.2024.106013

**Published:** 2024-11

**Authors:** Michael K. Lo, Shilpi Jain, Katherine A. Davies, Teresa E. Sorvillo, Stephen R. Welch, JoAnn D. Coleman-McCray, Payel Chatterjee, Anne L. Hotard, Troy O'Neal, Mike Flint, Huiwang Ai, Cesar G. Albariño, Jessica R. Spengler, Joel M. Montgomery, Christina F. Spiropoulou

**Affiliations:** a Centers for Disease Control and Prevention, Atlanta, GA, USA; b Emory National Primate Research Center, Emory Vaccine Center, Emory University, Atlanta, GA, USA; c U.S. Department of Agriculture, Agricultural Research Service, Zoonotic and Emerging Disease Research Unit, National Bio and Agro-Defense Facility, Manhattan, KS, USA; d University of Virginia, School of Medicine, Charlottesville, VA, USA

**Keywords:** Henipavirus, Nipah virus, Bangladesh, Reporter virus assay, ZsGreen fluorescent protein, Bioluminescent red protein, Antiviral screening, HSAEC1-KT, MRC-5, Syrian golden hamster

## Abstract

Nipah virus (NiV) causes near-annual outbreaks of fatal encephalitis and respiratory disease in South Asia with a high mortality rate (∼70%). Since there are no approved therapeutics for NiV disease in humans, the WHO has designated NiV and henipaviral diseases priority pathogens for research and development. We generated a new recombinant green fluorescent reporter NiV of the circulating Bangladesh genotype (rNiV-B-ZsG) and optimized it alongside our previously generated Malaysian genotype reporter counterpart (rNiV-M-ZsG) for antiviral screening in primary-like human respiratory cell types. Validating our platform for rNiV-B-ZsG with a synthetic compound library directed against viral RNA-dependent RNA polymerases, we identified a hit compound and confirmed its sub-micromolar activity against wild-type NiV, green fluorescent reporter, and the newly constructed bioluminescent red fluorescent double reporter (rNiV-B-BREP) NiV. We furthermore demonstrated that rNiV-B-ZsG and rNiV-B-BREP viruses showed pathogenicity comparable to wild-type NiV-B in the Syrian golden hamster model of disease, supporting additional use of these tools for both pathogenesis and advanced pre-clinical studies *in vivo*.

## Highlights

• Recombinant Nipah viruses expressing ZsGreen and BREP are suitable for antiviral screening.
• Library screening against rNiV-B-ZsG identified a hit compound with sub micromolar potency.
• rNiV-B-ZsG and rNiV-B-BREP reporter viruses retain wild-type pathogenicity in Syrian hamsters.

## 1 Introduction

Nipah virus (NiV) is a highly pathogenic bat-borne paramyxovirus that causes fatal encephalitis and respiratory disease in humans with high case fatality rates (40–75%). Since its initial outbreak in Malaysia, near-annual NiV spillovers into the human population have occurred in Bangladesh and, more recently, in India (Spiropoulou, 2019). NiV phylogenetically groups into two distinct genotypes historically associated with the initial Malaysian isolates (NiV-M; genotype M) or with the later Bangladesh isolates (NiV-B; genotype B) (Lo et al., 2012; Whitmer et al., 2021). Considering the current lack of approved therapeutics for NiV in humans and the documented spread of NiV through close contact with bodily fluids, the World Health Organization has included NiV in its R&D Blueprint list of epidemic threats (Nikolay et al., 2019; Plowright et al., 2019; WHO, 2019). We previously developed a ZsGreen1 (ZsG) fluorescent protein-expressing NiV based on the genotype M prototype sequence (rNiV-M-ZsG) for antiviral screening and for *ex vivo* imaging of infected tissues (Lo et al., 2014; Welch et al., 2020). In this study, we identified a novel antiviral compound through screening a compound library against a newly constructed genotype B-based ZsG reporter virus (rNiV-B-ZsG). We confirmed the activity of this compound against both wild-type viruses and another newly constructed genotype B recombinant virus that expresses a bioluminescent red protein (rNiV-B-BREP). Moreover, we showed that both reporter viruses are suitable for *in vitro* and *in vivo* use.

## 2 Material and methods

### 2.1 Biosafety and ethics

All work with infectious virus and infected animals was conducted in a biosafety level 4 (BSL-4) laboratory at the Centers for Disease Control and Prevention (CDC; Atlanta, USA) following established BSL-4 standard operating procedures approved by the Institutional Biosafety Committee. All recombinant virus work was approved by the CDC Institutional Biosafety Committee. All animal experiments were approved by the CDC Institutional Animal Care and Use Committee (3220SPEMULC) and performed in an AAALAC-International approved facility.

### 2.2 Cells & media

Vero (African green monkey kidney; ATCC CCL-81), Vero-E6 (ATCC-CRL-1586), and MRC-5 (human fetal lung fibroblast; ATCC CCL-171) cells were cultured and maintained according to manufacturer's recommended protocols. Baby hamster kidney cells stably expressing T7 polymerase (BSRT7/5) were maintained as previously described (Albariño et al., 2015). Human telomerase reverse-transcriptase- (hTERT) immortalized primary-like bronchial (HBEC3-KT; ATCC CRL-4051) and small airway (HSAEC1-KT; ATCC CRL-4050) epithelial cells were maintained in Airway Epithelial Cell Basal Media medium supplemented with Bronchial Epithelial Growth Kit. Microvascular hTERT-immortalized dermal endothelial cells (TIME) were maintained as previously described (Lo et al., 2021). All media were supplemented with antibiotics and antifungals. All cells were kept in humidified incubators at 37 °C and 5% CO_2_.

### 2.3 Viruses, cloning and rescue of recombinant viruses

Wild-type prototype Malaysian (wt NiV-M), Bangladesh (wt NiV-B), and rNiV-M-ZsG were grown as previously described (Lo et al., 2020a). Four DNA fragments were synthesized and combined to form the complete recombinant NiV (Bangladesh genotype; GenBank accession: AY988601.1; with correction in F gene coding sequence based on accession MK673564.1, as similarly done in (Griffin et al., 2019). The antigenome was cloned behind a T7 promoter followed by a hepatitis δ virus ribozyme and T7 terminator. The full-length clone was later modified to introduce different codon-optimized reporter genes (ZsG or BRET-based bioluminescent red protein [BREP]) (Tian et al., 2022) in-frame with the M gene ORF separated by the GGSG-P2A peptide sequence, as previously done for rNiV-M-ZsG using InFusion cloning technology (Takara Bio). Recombinant reporter-expressing NiVs were rescued as described previously (Lo et al., 2014) and sequenced by next-generation sequencing on a MiniSeq or iSeq platform (Illumina); no spurious changes were seen after passage. Reporter viruses were passaged at least 3 times and were confirmed to retain reporter gene expression. Recombinant mNeonGreen expressing SARS-CoV-2 was a kind gift from Dr. Pei-Yong Shi and was propagated as previously described (Lo et al., 2021).

### 2.4 TCID_50_ quantitation, multi-step growth curves, antiviral infectious yield assay, neutralization assay

Cell type-specific infectious titers were determined in triplicate for each virus stock in Vero, HBEC3-KT, HSAEC1-KT, TIME, and MRC-5 cells via 50% tissue culture infectious dose (TCID_50_) assays, as measured by visible cytopathic effect (CPE), reporter fluorescence, or antigen-specific indirect immune fluorescence (for MRC-5 cells). Multi-step time course growth curves for all wild-type and recombinant NiVs were performed in 12-well plates using multiplicity of infection (MOI) 0.025. At various timepoints post infection, supernatants were collected for TCID_50_ determination. Neutralization assays were conducted as previously described using monoclonal antibody 12B-2 (Absolute Antibody) or rabbit polyclonal serum directed against soluble Hendra virus G glycoprotein (Lo et al., 2020b). The lowest dilution in which 50% or 100% of all quadruplicate wells were negative for CPE were respectively designated as 50% and 100% neutralization titers (NT_50_ and NT_100_).

### 2.5 Reporter assays

All reporter-based assays were conducted in 96-well (Costar 3603) or 384-well (Agilent, 204628–100) plates with black walls and clear bottoms. All CPE and cell viability assays were conducted in either 96-well or 384-well white plates (Costar 3917, 3570).

#### 2.5.1 Fluorescence intensity and image-based assays

Fluorescence intensity of ZsG and mScarlet-I reporters was assayed in an H1 Synergy plate reader (Agilent) using the bottom read function with excitation (ex)/emission (em) wavelengths of 488_em_/518_ex_ (for ZsG) and 568_ex_/598_em_ (for mScarlet-I) (gain setting = 75). Cytation 3 imagers (Agilent) were used for all image-based assays, as previously described (Lo et al., 2018, 2020a). Fluorescence intensity measures from mock-infected well images were set as baseline cutoffs for detection of positive vs. negative signal for determining fluorescence intensity and confluence. For red fluorescence imaging, an RFP filter cube was used. All image-based calculations were based on whole wells (comprised of 6 montaged images taken at 2.5 × magnification).

#### 2.5.2 Luminescence-based assays

Nano-Glo® and CellTiter-Glo® 2.0 assay reagents (Promega) were used according to manufacturer's protocols to respectively measure bioluminescence from Nano luciferase-based BREP reporter viruses and cellular ATP levels (Lo et al., 2021 MSpectrum). For BREP reporter virus bioluminescence optimization, infected cells treated with Nano luciferase substrate were transferred to solid white plates and read for luminescence signal.

### 2.6 Antiviral compound library screen

The Viral RNA-Dependent RNA Polymerases Targeted Library (1134 unique compounds at the time of purchase) was procured from Otava Chemicals (Viral RNA-dependent RNA Polymerases Targeted Library (otavachemicals.com)). Compounds were dissolved in dimethyl sulfoxide and stored in 384-well Echo acoustic liquid handler-compatible source plates. Compounds were dispensed into 384-well black/clear bottom plates previously seeded with HSAEC1-KT cells (∼2500–3000 cells/well overnight) for 1–2 h, and then infected with rNiV-B-ZsG (MOI = 0.25) in a total reaction volume of 30 μL. Reporter signal for the screen was read at 72 h post-infection.

### 2.7 RNA extraction, RNA-seq library construction, sequencing, and data analysis

HSAEC1-KT and MRC-5 cells were cultured in 12-well plates and inactivated using TriPURE reagent (Millipore Roche). RNA was extracted using the Direct-ZOL kit according to manufacturer's instructions (Zymo Research). NEBNext® Ultra™ II Directional RNA Library Prep Kit for Illumina® was used for RNA library construction. RNA libraries were sequenced on a Novaseq X instrument PE150, 40M total reads (20M in each direction) (Omega Bioservices, Norcross, USA). Data were analyzed by ROSALIND® (https://rosalind.bio/), with HyperScale architecture developed by ROSALIND, Inc. (San Diego, CA). Reads were trimmed using Cutadapt (Martin, 2011). Quality scores were assessed using FastQC (http://www.bioinformatics.babraham.ac.uk/projects/fastqc). Reads were aligned to the *Homo sapiens* genome build hg38 using STAR (Dobin et al., 2013). Individual sample reads were quantified using HTseq4 and normalized via relative log expression using the DESeq2 R library (Love et al., 2014). Read distribution percentages, violin plots, identity heatmaps, and sample MDS plots were generated as part of the QC step using RSeQC (Wang et al., 2012). DEseq2 was also used to calculate fold changes and p-values and perform optional covariate correction.

### 2.8 Hamster studies

Groups of ten 5-week-old HsdHan: AURA golden Syrian hamsters (Envigo; catalog no. 8902F or 8902M, 5 male and 5 female per group) were inoculated intranasally (100 μL divided bilaterally) with a target dose of ∼1 × 10^6^ TCID_50_ wt NiV-B (5.95 × 10^5^ TCID_50_), rNiV-B-ZsG (1.13 × 10^6^ TCID_50_), or rNiV-B-BREP (2.72 × 10^6^ TCID_50_). Hamsters were kept in a climate-controlled laboratory with a 12-h day/night cycle, provided Teklad global 18% protein rodent diet (Envigo) and water *ad libitum*, and group-housed on corn cob bedding (Bed-o'Cobs, ¼ in.; Anderson Lab Bedding, Maumee, USA) with cotton nestlets and crinkle paper in an isolator caging system (Tecniplast Green Line GR900 caging, West Chester, USA) with HEPA-filtered inlet and exhaust air supply. Microchip transponders (BMDS; IPTT-300) were placed subcutaneously in the interscapular region 4 days before infection (−4 dpi) for individual identification and body temperature assessment. Baseline weights were taken −1 dpi, and weight changes, body temperatures, and clinical signs were assessed daily. Clinical signs were scored as previously described (Welch et al., 2022). Hamsters were euthanized with isoflurane vapor when they met euthanasia criteria (score ≥10) or at study completion (28 dpi).

#### 2.8.1 Tissue RNA analysis

RNA was extracted from tissue samples (liver, spleen, reproductive organs [testes/ovary], kidney, heart, lung, eye, brain) homogenized in 1 mL MagMax lysis buffer concentrate, or from 50 μL whole blood collected in Dipotassium EDTA and added to 500 μL MagMax lysis buffer concentrate. Extractions were performed using the MagMax-96 Total RNA isolation kit (Thermo-Fisher) on the KingFisher Apex Purification System (Thermo-Fisher). Samples were treated with DNase-1 (Lucigen) and eluted into 75 μL elution buffer. Tissue RNA levels were obtained using RT-qPCR assay targeting the NiV N gene sequence (NiV forward, 5′-CTGGTCTCTGCAGTTATCACCATCGA-3′; NiV reverse, 5′-ACGTACTTAGCCCATCTTCTAGTTTCA-3′; and NiV probe, 5′-(6-FAM)-CAGCTCCCG-(ZEN™)-ACACTGCCGAGGAT-(IBFQ)-3′; all from IDT) with levels normalized using the validated reference genes Hrpt and Ppia (Davies et al., 2023). Viral target assays were performed in singleplex, and housekeeping assays in duplex. Genome copy numbers were determined using standards prepared from Ultramer RNA Oligonucleotide (IDT) containing the NiV-N amplicon sequence.

### 2.9 Statistical analysis

Statistical analyses were performed using Prism 9 software (GraphPad Software). Signal-to-background ratio (S/B), percent coefficient of variation (%CV), Z′-scores, 50% viability/cytotoxicity (CC_50_), 50% effective concentration (EC_50_), and selective indices (SI) were calculated as previously described (Lo et al., 2014).

## 3 Results and discussion

### 3.1 Recombinant ZsG-expressing NiV genotype Bangladesh and Malaysia exhibit phenotypes similar to wild-type virus in vero cells

**Fig. 1:**
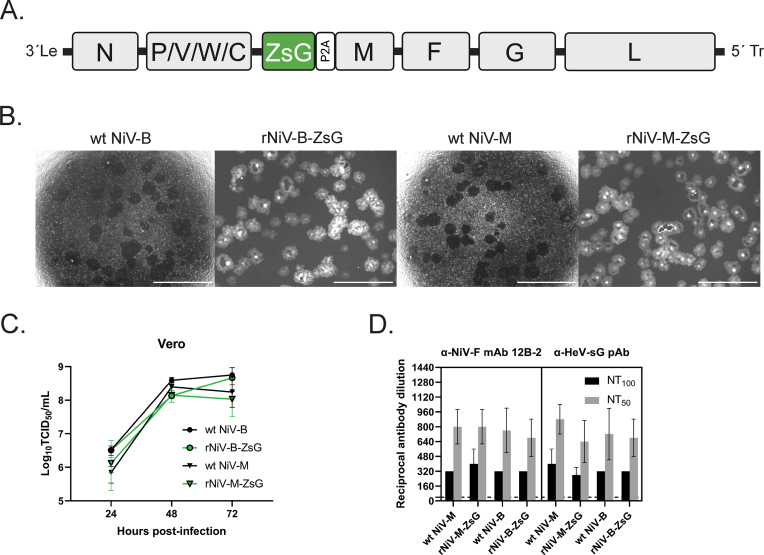
Recombinant Malaysia and Bangladesh genotype Nipah viruses (NiV) expressing ZsGreen1 (rNiV-M-ZsG, rNiV-B-ZsG) have growth and antigenic phenotypes comparable to wild-type virus (wt-NiV-M, wt-NiV-B). (A) Schematic representation of the negative-sense genome of the recombinant NiVs expressing ZsG, which is encoded within the M gene separated by the cleavage/ribosomal skipping 2A peptide sequence from porcine teschovirus-1. (B) Low magnification (2 × ) light and fluorescence micrographs of Vero cells infected with wild-type NiV or rNiV-M-ZsG/rNiV-B-ZsG, respectively, 24 h post infection (hpi). Dark areas in the monolayer seen on light micrographs indicate syncytia formation, whereas gray-white patches in the fluorescence micrographs indicate ZsG signal within syncytia. White bars indicate 2000 μm. (C) Multi-step growth curves. Vero cells were infected with wt-NiV-M, wt-NiV-B, rNiV-M-ZsG, or rNiV-B-ZsG at MOI = 0.025 for 1 h. After removal of NiV inoculum, cells were washed once with phosphate-buffered saline, and replenished with growth media. At 24, 48, and 72 hpi, cell supernatants were harvested and serially diluted 10-fold for inoculating Vero cells to assess virus yield by 50% tissue culture infectious dose (TCID_50_) assay. (D) Equivalent neutralization of wild-type and recombinant ZsG-expressing NiVs by fusion glycoprotein (12B-2) and attachment glycoprotein (HeV-sG)-specific antibodies. 125 TCID_50_ of each virus were pre-incubated for 1 h at room temperature with serial dilutions of a monoclonal antibody against NiV fusion (F) protein (12B-2) or cross-reactive polyclonal antiserum generated against Hendra virus soluble attachment glycoprotein (HeV-sG). Virus-containing solutions were overlaid directly onto Vero cells. Six days post infection, TCID_50_ assays were conducted to determine reciprocal 50% and 100% virus neutralization titers (NT_50_, NT_100_). Error bars indicate standard deviation of the mean of 3 independent experiments conducted with 4 technical replicates.

We constructed and rescued rNiV-B-ZsG, a recombinant reporter NiV Bangladesh virus encoding a ZsGreen1 fluorescent protein in frame with the M gene separated by the porcine teschovirus 2A peptide cleavage/ribosomal skipping sequence, as done previously with our Malaysia genotype recombinant ZsG reporter NiV (rNiV-M-ZsG; previously designated as rNiV-GFP2AM, NiV-M/ZsG) (Lo et al., 2014; Welch et al., 2020) (Fig. 1A). Successful virus rescue hinged upon correcting the fusion (F) gene coding sequence of the full-length antigenomic cDNA based on the initial prototype NiV-B sequence (GenBank accession AY988601). For this, we used the F gene sequence of a re-sequenced passage 2 stock of the prototype Bangladesh isolate (GenBank Accession MK673564) (Griffin et al., 2019; Whitmer et al., 2021). We conducted multi-step growth curves (MOI = 0.025) to compare growth phenotypes of these reporters in Vero cells alongside wild-type Malaysia and Bangladesh (wt NiV-M, wt NiV-B) viruses, and observed no apparent differences in virus-induced syncytium sizes 24 h post infection (hpi) (Fig. 1B). Both viral strains grew to high infectious titers with no consistent inter-strain-related statistical differences. Infectious titers for wt-NiV-M and rNiV-M-ZsG began to decline by 72 hpi, whereas wt-NiV-B and rNiV-B-ZsG either maintained or reached peak titers 72 hpi (Fig. 1C, Supplemental Fig. 1). We then conducted neutralization tests with the same viruses using a monoclonal antibody against NiV F glycoprotein (12B-2) (Dang et al., 2019) and a polyclonal serum against soluble Hendra virus attachment glycoprotein (G) with known cross-reactivity to NiV G (Lo et al., 2020b); no significant differences in 50% or 100% virus neutralization titers were observed (Fig. 1D).

### 3.2 Differences in NiV infectivity in human small airway and fetal lung fibroblast cell lines correlate with EphrinB2 mRNA expression levels

**Fig. 2:**
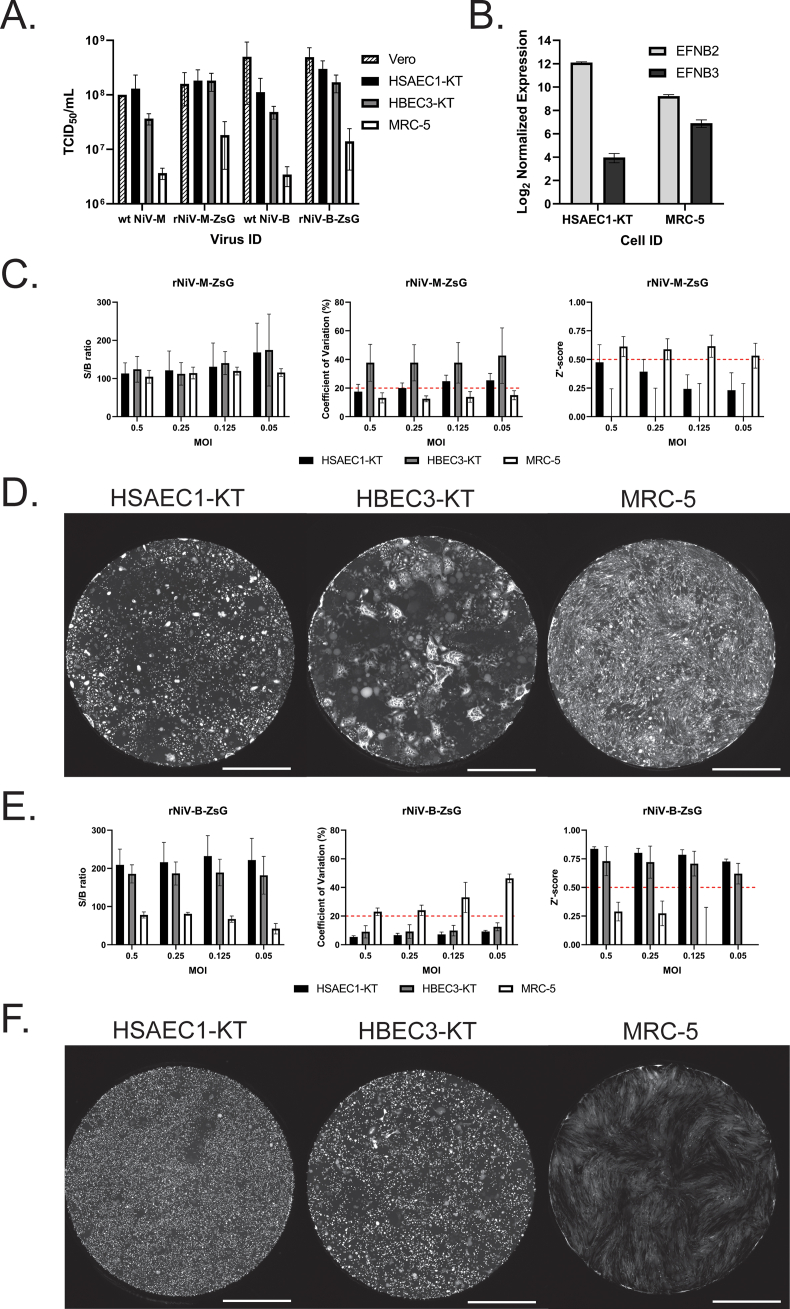
Characterization and optimization of human respiratory cell lines for antiviral screening indicate virus strain-specific phenotypes. (A) Comparative infectivity of wt-NiV-M, rNiV-M-ZsG, wt-NiV-B, and rNiV-B-ZsG in Vero, HSAEC1-KT, HBEC3-KT, and MRC-5 cell lines. Error bars indicate standard deviation of the mean of 3 independent titrations of each respective virus stock. (B) Normalized mRNA transcript levels of Ephrin B2 and Ephrin B3 in uninfected HSAEC1-KT and MRC-5 cells. Error bars indicate standard deviation of the mean of 4 independent samples. Signal-to-background (S/B) ratios (left panel), % coefficient of variation (%CV; center panel), and Z′-scores (right panel) of (C) rNiV-M-ZsG-infected and (E) rNiV-B-ZsG-infected HSAEC1-KT (black bars), HBEC3-KT (gray bars), or MRC-5 (white bars) cells at indicated MOI in 96-well plates 72 hpi. Error bars indicate standard deviation of the mean of at least 4 independent experiments. Whole-well montaged fluorescence micrographs of indicated cell lines infected with (D) rNiV-M-ZsG or (F) rNiV-B-ZsG 72 hpi. White bar indicates 2000 μm in length.

Given the phenotypic liabilities of cell lines with aberrant expression of drug efflux transporters or compromised innate immunity (e.g., Vero and HEK-293T cells) (De Rosa et al., 2004; Rausell et al., 2016), we evaluated and compared three primary-like human respiratory cell lines for suitability in antiviral screening with rNiV-M-ZsG and rNiV-B-ZsG. These cell lines – human bronchial (HBEC3-KT) and small airway epithelial cells (HSAEC1-KT) immortalized with hTERT (Nakauchi et al., 2019), and human diploid fetal lung fibroblasts (MRC-5) (Jacobs et al., 1970)– physiologically represent early targets of NiV infection. To ensure use of equivalent MOI in our comparisons, we determined cell line-specific infectious titers for each virus in the 3 human cell lines by TCID_50_ assay and compared them to their respective titers in Vero cells. Of the 3 human cell lines, HSAEC1-KT cells had equivalent (wt-NiV-M, rNiV-M-ZsG) or near-equivalent (wt-NiV-B, rNiV-B-ZsG) infectivity as Vero cells, whereas MRC-5 cells showed approximately 1–1.5 log_10_ lower infectivity across all viruses used (Fig. 2A). The relatively high susceptibility of HBEC3-KT and HSAEC1-KT cells to NiV infection is similar to findings with primary human bronchial and small airway epithelial cells (Escaffre et al., 2013). We sequenced mRNA of uninfected HSAEC1-KT and MRC-5 cells to determine and compare relative mRNA transcript levels of the two known functional receptors for NiV infection, EphrinB2 (EFNB2) and EphrinB3 (EFNB3; Fig. 2B) (Negrete et al., 2005, 2006). Both cell lines expressed higher levels of EFNB2 transcripts than of EFNB3, suggesting EFNB2 as the primary receptor used for virus entry. Accordingly, the approximately 8-fold greater mRNA levels of EFNB2 in HSAEC1-KT cells than in MRC-5 cells correlate with higher NiV infectivity of HSAEC1-KT cells.

### 3.3 Optimization of reporter expression by rNiV-M-ZsG and rNiV-B-ZsG for antiviral screening in human respiratory cell lines indicates divergent genotype-specific phenotypes

We infected HSAEC1-KT, HBEC3-KT, and MRC-5 cells with rNiV-M-ZsG or rNiV-B-ZsG at 0.5, 0.25, 0.125, or 0.05 MOI in 96-well plates for 72 h to evaluate their respective suitability for antiviral screening assays. The criteria used to evaluate assay quality for each cell type were ZsG reporter S/B ratio, %CV of positive control ZsG reporter signal, and Z′-score (Zhang et al., 1999).

The S/B ratio observed for rNiV-M-ZsG for both HSAEC1-KT and HBEC3-KT cells ranged between 100 and 150, with high % CV. Z′-scores were <0.5 across all tested MOI in HSAEC1-KT and HBEC3-KT cells, indicating their unsuitability for antiviral screening. Infected MRC-5 cells, however, showed remarkably consistent S/B ratios with comparably little variability across all MOIs tested, and had a Z′-score >0.5, indicating this cell line's potential utility for antiviral screening (Fig. 2C–Table 1). Upon comparing whole-well fluorescence images of the three infected cell lines, we observed heterogeneous ZsG signal distribution due to extensive syncytia formation and CPE in both HSAEC1-KT and HBEC3-KT cells. CPE was less pronounced in MRC-5 cells, resulting in relatively homogeneous reporter signal distribution across the monolayer (Fig. 2D). Despite attempts to measure ZsG reporter signal at earlier time points to reduce the influence of syncytia formation in HSAEC1-KT and HBEC3-KT cells, Z′-scores never reached values > 0.5 (data not shown).

**Table 1:** Optimization of recombinant ZsG fluorescent reporter Nipah viruses (rNiV-M-ZsG or rNiV-B-ZsG) for antiviral screening in MRC-5 or HSAEC1-KT cells 72 h post infection.

MRC-5 (rNiV-M-ZsG infection)				
Assay format	MOI	S/B ratio	CV (%)	Z′-score
96-well	0.5	105 ± 17	13 ± 4	0.61 ± 0.09
	0.25	114 ± 15	13 ± 2	0.59 ± 0.09
	0.125	120 ± 10	14 ± 4	0.62 ± 0.10
	0.05	116 ± 10	15 ± 3	0.53 ± 0.11
384-well	0.25	77 ± 7	15 ± 1	0.53 ± 0.04
**HSAEC1-KT (rNiV-B-ZsG infection)**				
**Assay format**	**MOI**	**S/B ratio**	**CV (%)**	**Z′-score**
96-well	0.5	209 ± 42	5 ± 1	0.84 ± 0.02
	0.25	216 ± 52	7 ± 1	0.80 ± 0.04
	0.125	232 ± 54	7 ± 2	0.79 ± 0.04
	0.05	221 ± 57	9 ± 1	0.73 ± 0.02
384-well	0.25	171 ± 17	10 ± 3	0.70 ± 0.08

MOI, multiplicity of infection; S/B, signal-to-background ratio; CV, coefficient of variation. Mean values with ±standard deviation values were derived from a minimum of 5 independent experiments for each MOI tested.

In contrast to rNiV-M-ZsG, HSAEC1-KT and HBEC3-KT cells infected with rNiV-B-ZsG had higher S/B ratios (>200 in HSAEC1-KT cells), lower %CV values (<10% in HSAEC1-KT), and more robust Z′-scores (>0.6 in HSAEC1-KT cells) across all tested MOI. Furthermore, MRC-5 cells infected with rNiV-B-ZsG showed lower mean S/B ratios (50–80) and higher %CV values (>20%) that inversely correlated with MOI, and suboptimal Z′-scores (<0.3) across all tested MOI (Fig. 2E–Table 1). Interestingly, ZsG reporter distribution patterns in rNiV-B-ZsG-infected HSAEC1-KT cells were highly homogenous, showing fewer dark patches of CPE than observed in rNiV-M-ZsG-infected cells (Fig. 2F, left panel). A similar effect was observed in rNiV-B-ZsG-infected HBEC3-KT, but not to the extent observed in HSAEC1-KT cells (Fig. 2F, middle panel). rNiV-B-ZsG-infected MRC-5 cells also showed a phenotype distinct from that of rNiV-M-ZsG infection, with infected cells showing a larger range of fluorescence intensities and visibly larger syncytia (Fig. 2F, right panel). Although a comprehensive comparative analysis of the factors underlying the observed phenotypic differences between rNiV-B-ZsG and rNiV-M-ZsG is outside the scope of this study, these observations underscore the importance of investigating genotype-specific differences in physiologically relevant cell types that closely approximate primary target cells (Ang et al., 2022; DeBuysscher et al., 2021; Escaffre et al., 2013).

### 3.4 Optimization and validation of rNiV-B-ZsG infection in 384-well format against a synthetic compound library identified a hit compound with sub-micromolar potency

**Fig. 3:**
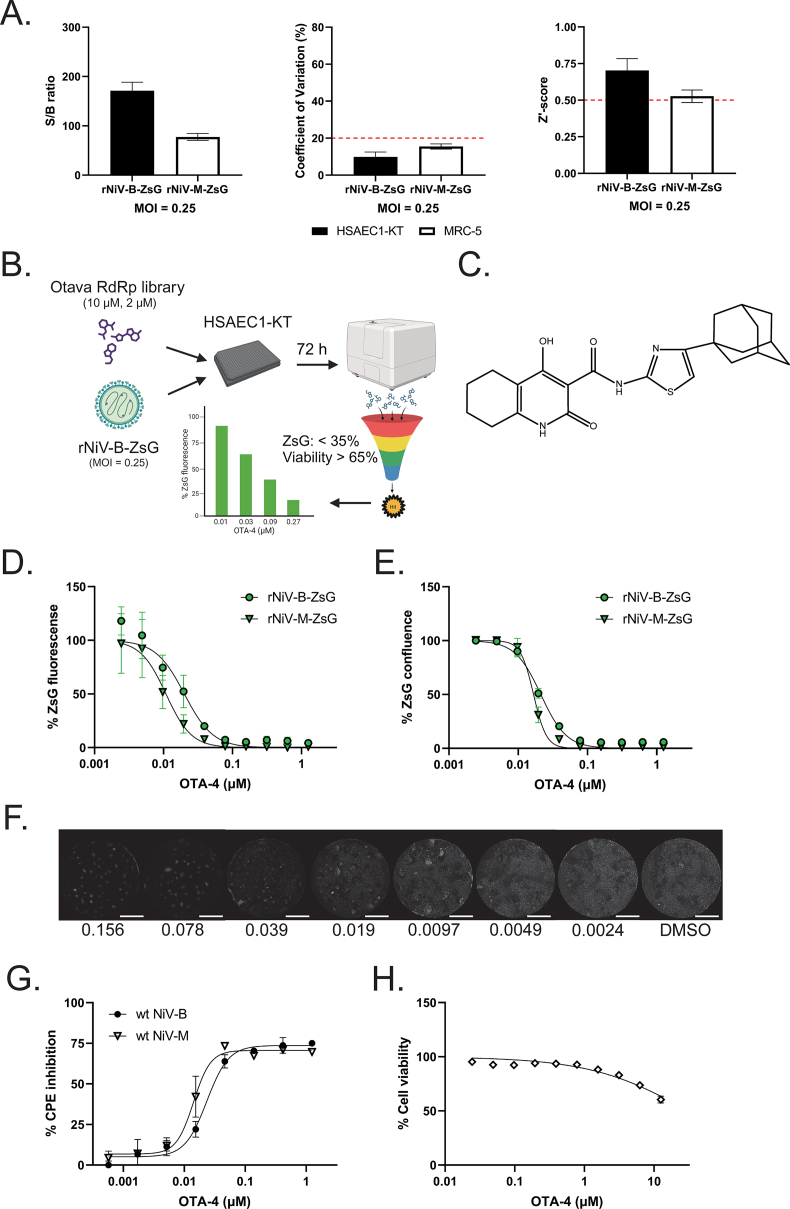
Optimization and validation of rNiV-B-ZsG antiviral screening platform in 384-well plate format against a synthetic compound library identifies a potent antiviral hit compound. (A) S/B ratio (left panel), %CV (center panel), and Z′-scores (right panel) of rNiV-B-ZsG-infected HSAEC1-KT cells or rNiV-M-ZsG-infected MRC-5 cells at MOI = 0.25 in 384-well plate format 72 hpi. Error bars indicate standard deviation of the mean of at least 6 independent experiments. (B) Schematic of antiviral library screen workflow. The Otava RNA-dependent RNA polymerase-directed library was screened at 2 concentrations, and only compounds that reduced reporter ZsG signal to <35% and had viability of >65% at both concentrations were deemed a hit for further dose-response confirmation. (C) Structure of antiviral hit compound OTA-4 (details of chemical name in the text and methods). Representative OTA-4 antiviral dose-response curves against rNiV-B-ZsG-infected or rNiV-M-ZsG-infected HSAEC1-KT cells were constructed by (D) measuring ZsG fluorescence intensity and by (E) image-based ZsG fluorescence confluence 72 hpi. Error bars indicate standard deviation of the mean of 4 biological replicates. (F) Representative whole-well montaged fluorescence micrographs of rNiV-B-ZsG-infected HSAEC1-KT cells treated with indicated amounts of OTA-4 (μM). White bars indicate 2000 μm. (G) Representative OTA-4 antiviral dose-response counter screen against wt-NiV-B-induced and wt-NiV-M-induced CPE 72 hpi. Error bars indicate standard deviation of the mean of 3 biological replicates. (H) Dose-response evaluation of OTA-4 treatment on HSAEC1-KT cell viability. Error bars indicate standard error of the mean across 7 independent experiments conducted in quadruplicate.

**Fig. 4:**
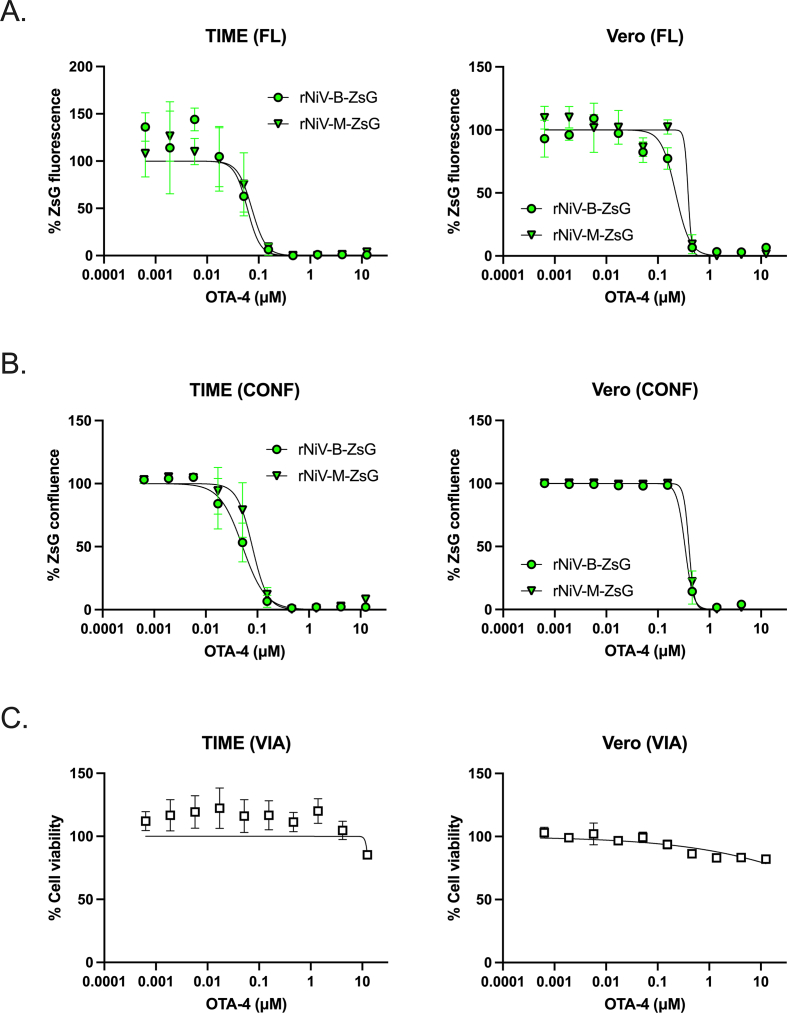
OTA-4 inhibits ZsG-reporter NiVs in hTERT-immortalized microvascular endothelial (TIME) and Vero cells. Representative OTA-4 antiviral dose-response curves against rNiV-B-ZsG-infected (green circles) or rNiV-M-ZsG-infected (green triangles) TIME cells (left column) or Vero cells (right column) were conducted by measuring (A) ZsG fluorescence intensity and (B) image-based ZsG fluorescence confluence 72 hpi. (C) Dose-response evaluation of OTA-4 treatment on TIME and Vero cell viability. Error bars indicate standard deviation of the mean of 3 biological replicates conducted in at least 2 independent experiments.

We then infected HSAEC1-KT with NiV-B-ZsG and MRC-5 cells with rNiV-M-ZsG (MOI = 0.25) in a higher throughput 384-well plate format, and observed S/B ratios, %CV values, and Z′-scores approximating those derived from 96-well plate infections (Fig. 3A, Table 1). Since rNiV-B-ZsG infection in HSAEC1-KT cells yielded more robust values across our quality criteria, we used this 384-well platform to screen a synthetic library of compounds designed against viral RNA-dependent RNA polymerases. We screened 10 μM and 2 μM of each library compound in triplicate against rNiV-B-ZsG and conducted accompanying compound cytotoxicity assays in duplicate (Fig. 3B). We identified one molecule – N-[4-(adamantan-1-yl)-1,3-thiazol-2-yl]-4-hydroxy-2-oxo-1,2,5,6,7,8-hexahydroquinoline-3-carboxamide; referred to as OTA-4 – that met our hit criteria of reducing ZsG reporter signal to <35% and maintaining >65% cell viability across both concentrations tested (Fig. 3C) (antiviral hit rate = 0.009%, hit confirmation rate = 100%). We conducted OTA-4 dose-response experiments in HSAEC1-KT cells infected with rNiV-B-ZsG or rNiV-M-ZsG using our standard fluorescence intensity assay (Fig. 3D), as well as a whole-well image-based reporter confluence assay that determines the proportion of a well positive for ZsG signal (Fig. 3E and F). OTA-4 inhibited both viruses in both assays in a dose-dependent manner, with EC_50_ in the low double-digit nanomolar range (Table 2). We confirmed the antiviral activity of OTA-4 in an orthogonal CPE assay and showed dose-dependent reduction of CPE against both wt NiV-M and wt NiV-B (Fig. 3G–Table 2). Furthermore, OTA-4 showed minimal cytotoxicity in uninfected cells (Fig. 3H). To ensure that the observed inhibition was not cell line-specific, we tested OTA-4 against rNiV-B-ZsG and rNiV-M-ZsG in both TIME and Vero cells, observing dose-dependent inhibition in both cell lines with sub-micromolar EC_50_ concentrations that were 3-fold (TIME) to 10-fold (Vero) higher than those observed in HSAEC1-KT cells (Fig. 4, Table 3). To confirm virus-specific activity of OTA-4, we evaluated OTA-4 against a reporter SARS-CoV-2 virus expressing mNeonGreen and observed no antiviral activity across all concentrations tested in Vero cells (Table 3, data not shown).

**Table 2:** Mean antiviral activity and selective indices of OTA-4 against wild-type and recombinant reporter NiVs in HSAEC1-KT cells.

Virus ID	Assay	Cell ID		
		HSAEC1-KT (CC_50_ > 12.5 μM)		
		EC_50_	EC_90_	SI
rNiV-B-ZsG	FL	0.017 ± 0.002	0.057 ± 0.006	>735
	CONF	0.022 ± 0.003	0.049 ± 0.006	>568
rNiV-M-ZsG	FL	0.011 ± 0.001	0.028 ± 0.005	>1136
	CONF	0.016 ± 0.002	0.027 ± 0.003	>781
wt NiV-B	CPE	0.021 ± 0.002	0.049 ± 0.01	>595
wt NiV-M	CPE	0.014 ± 0.002	0.025 ± 0.004	>892
rNiV-B-BREP	FL	0.013 ± 0.002	0.020 ± 0.003	>961
	CONF	0.015 ± 0.002	0.026 ± 0.006	>833
	LUM	0.011 ± 0.003	0.020 ± 0.002	>1136
	VTR	0.016	0.023	>781

CC_50_, 50% cell cytotoxicity concentration; EC_50_, 50% effective inhibition concentration; EC_90_, 90% effective inhibition concentration; SI, selective index (EC_50_/CC_50_); FL, fluorescence intensity assay; CONF, image-based fluorescence confluence assay; LUM, bioluminescence assay; CPE, cytopathic effect; VTR, virus titer reduction. Mean values with ±standard deviation values were derived from a minimum of 3 independent experiments performed in biological quadruplicate.

**Table 3:** Mean antiviral activity and selective indices of OTA-4 against recombinant reporter viruses in TIME and Vero cells.

Virus family	Virus ID	Assay	Cell ID					
			TIME (CC_50_ > 12.5 μM)			Vero (CC_50_ > 12.5 μM)		
			EC_50_	EC_90_	SI	EC_50_	EC_90_	SI
*Paramyxoviridae*	rNiV-B-ZsG	FL	0.060 ± 0.01	0.23 ± 0.1	>208	0.18 ± 0.03	0.49 ± 0.03	>69
		CONF	0.10 ± 0.03	0.19 ± 0.05	>125	0.21 ± 0.01	0.43 ± 0.02	>59
	rNiV-M-ZsG	FL	0.055 ± 0.02	0.13 ± 0.03	>227	0.24 ± 0.05	0.41 ± 0.2	>52
		CONF	0.077 ± 0.01	0.13 ± 0.05	>162	0.32 ± 0.07	0.50 ± 0.1	>39
*Coronaviridae*	rSARS-CoV-2 mNG	FL	NT	NT	N/A	No inh.	No inh.	N/A
		CONF	NT	NT	N/A	No inh.	No inh.	N/A

CC_50_, 50% cell cytotoxicity concentration; EC_50_, 50% effective inhibition concentration; EC_90_, 90% effective inhibition concentration; SI, selective index (EC_50_/CC_50_); FL, fluorescence intensity assay; CONF, image-based fluorescence confluence assay; NT, not tested; N/A, not applicable; No inh., no inhibition. Mean values with ±standard deviation values were derived from a minimum of 3 independent experiments performed in biological quadruplicate.

### 3.5 Characterization and antiviral optimization of rNiVs expressing a double reporter BRET-based bioluminescent red protein (BREP)

**Fig. 5:**
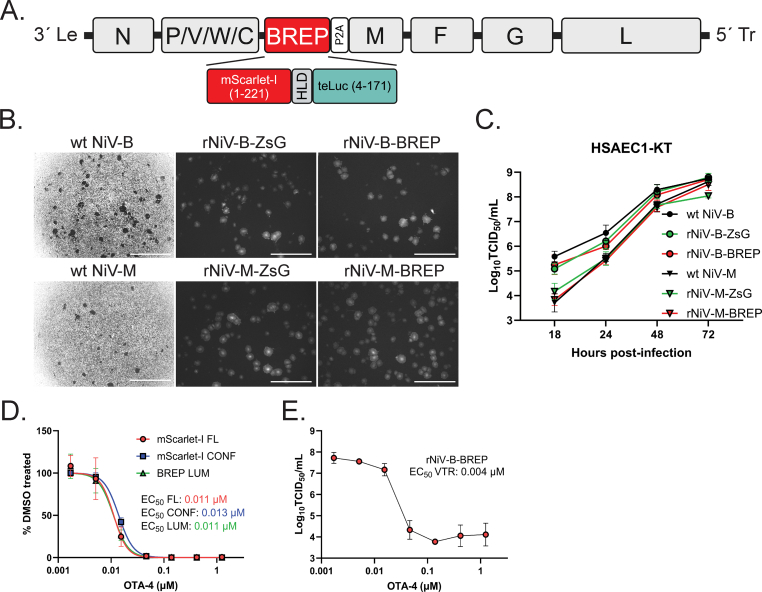
Construction, characterization, and antiviral optimization of recombinant bioluminescent red protein (BREP)-expressing NiV. (A) Schematic representation of the negative-sense genome of recombinant NiVs expressing the reporter BREP, which is encoded within the M gene separated by the cleavage/ribosomal skipping 2A peptide sequence from porcine teschovirus-1. (B) Low magnification (2 × ) light and fluorescence micrographs of HSAEC1-KT cells infected with wild-type, recombinant ZsG-expressing, or recombinant BREP-expressing NiVs (rNiV-B-BREP, rNiV-M-BREP), respectively, 18 hpi. Darkened holes seen in the monolayer in light micrographs indicate syncytia formation, whereas gray-white patches in the fluorescence micrographs indicate ZsG or BREP fluorescence signal within syncytia. White bars indicate 2000 μm. (C) Multi-step growth curves. HSAEC1-KT cells were infected with wt NiV-M, wt NiV-B, rNiV-M-ZsG, rNiV-B-ZsG, rNiV-M-BREP, or rNiV-B-BREP at MOI 0.025 for 1 h. After removal of inoculum, cells were washed once with phosphate-buffered saline, and replenished with growth media. At 18, 24, 48, and 72 hpi, cell supernatants were harvested and subjected to TCID_50_ assay to assess virus yield. (D) OTA-4 antiviral dose-response counter screen against rNiV-B-BREP using 3 reporter-based assay readouts at 48 hpi: 1) fluorescence (red circles), 2) image-based fluorescence confluence (blue squares), and 3) bioluminescence (green triangles). (E) OTA-4 efficiently reduces rNiV-B-BREP infectious virus yield by > 3.5 log_10_TCID_50_. HSAEC1-KT cells were pre-treated with serial dilutions of OTA-4 for 1 h, and then infected with rNiV-B-BREP (MOI = 0.1). At 48 hpi, infectious yield in collected cell supernatant was measured by TCID_50_ assay.

To develop additional orthogonal antiviral counter screens and to enable future *in vivo* imaging studies of NiV-infected animals, we constructed recombinant Malaysia and Bangladesh NiVs expressing a codon-optimized BRET-based bioluminescent red protein (BREP) reporter fusion construct comprised of an mScarlet-I red fluorescence protein linked to a teal-emitting Nano luciferase (teLuc) (Tian et al., 2022) (Fig. 5A). Both rNiV-B-BREP and rNiV-M-BREP viruses showed growth characteristics similar to their wild-type and recombinant ZsG-expressing counterparts in HSAEC1-KT cells (Fig. 5B), with Bangladesh viruses growing to higher infectious titers than Malaysia viruses 18–48 hpi (Fig. 5C, Supplemental Fig. 2). Assay optimization for rNiV-B-BREP in HSAEC1-KT cells showed that mScarlet-I reporter fluorescence and BREP-based bioluminescence were suitable for antiviral screening using multiple MOIs (Table 4). We counter screened OTA-4 against rNiV-B-BREP in HSAEC1-KT cells using three assay readouts: mScarlet-I fluorescence intensity, image-based mScarlet-I reporter confluence, and BREP luminescence 48 hpi. The corresponding EC_50_ values obtained from these assays correlated not only to one another but also to the values derived from ZsG reporter and wild-type CPE assays (Fig. 5D–Table 2). We furthermore conducted an infectious yield reduction assay using rNiV-B-BREP and OTA-4 and observed a dose-dependent decrease (>3.5 log_10_ TCID_50_/mL) in virus titer (Fig. 5E). Investigation of the mechanism of action and the broad-spectrum potential of OTA-4 activity is ongoing, as it has yet to be determined whether OTA-4 is a direct-acting or a host-directed antiviral.

**Table 4:** Optimization of mScarlet-I fluorescence and BREP-based luminescence expression in rNiV-B-BREP-infected HSAEC1-KT cells 72 h post infection.

mScarlet-I fluorescence				
Assay format	MOI	S/B ratio	CV (%)	Z′-score
96-well	0.5	207 ± 11	5 ± 1	0.84 ± 0.04
	0.25	229 ± 14	8 ± 1	0.77 ± 0.04
	0.125	224 ± 26	11 ± 3	0.68 ± 0.10
	0.05	239 ± 39	11 ± 2	0.66 ± 0.06
	0.025	216 ± 25	12 ± 3	0.63 ± 0.09
	0.0125	219 ± 32	16 ± 4	0.52 ± 0.12
384-well	0.25	203 ± 36	9 ± 3	0.73 ± 0.08
	0.1	219 ± 33	10 ± 2	0.69 ± 0.04
**BREP luminescence**				
**Assay format**	**MOI**	**S/B ratio**	**CV (%)**	**Z′-score**
96-well	0.125	1023 ± 93	5 ± 1	0.86 ± 0.04
	0.05	1125 ± 61	6 ± 1	0.83 ± 0.03
	0.025	1204 ± 104	6 ± 1	0.81 ± 0.02
	0.0125	1901 ± 163	10 ± 5	0.69 ± 0.2

MOI, multiplicity of infection; S/B, signal-to-background ratio; CV, coefficient of variation. Mean values with ±standard deviation values were derived from a minimum of 4 independent experiments for each MOI tested.

### 3.6 rNiV-B-ZsG and rNiV-B-BREP retain wild-type pathogenicity in syrian golden hamsters

**Fig. 6:**
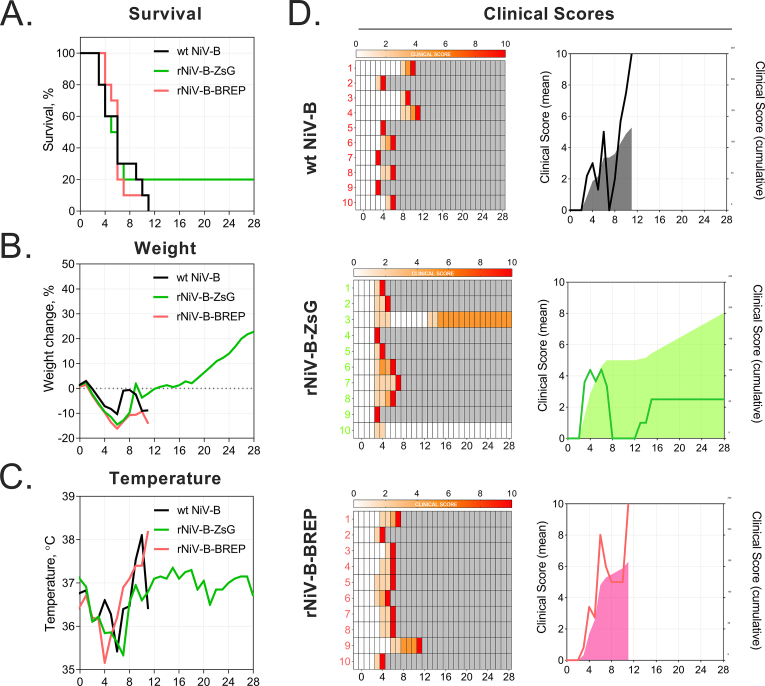
Recombinant reporter Bangladesh genotype NiVs retain similar pathogenicity to wild-type virus in Syrian golden hamster disease model. Mean (A) survival, (B) weight, and (C) temperature for groups of Syrian golden hamsters (N = 10 per group) inoculated intranasally with 10^6^ TCID_50_ of wt-NiV-B (black line), rNiV-B-ZsG (green line), or rNiV-B-BREP (red line). (D) Individual (left panels), mean, and cumulative clinical scores (right panels) for indicated infected groups. Increases in clinical scores of individual animals correspond with increasing intensity of red colored boxes (center panels) as indicated on the color scale, with solid red indicating animal was removed from study. Gray-shaded boxes indicate deceased animal no longer being scored. Mean and cumulative clinical scores are indicated respectively by lines (left y-axis) and area of shaded color (right y-axis).

We compared the *in vivo* pathogenicity of rNiV-B-ZsG and rNiV-B-BREP with that of wt NiV-B in the Syrian golden hamster model (Lo et al., 2020b) infected intranasally (target dose: 10^6^ TCID_50_). Apart from two rNiV-B-ZsG-infected animals that survived challenge (one of which displayed sustained clinical signs until study termination), temperatures, weights, clinical scores, median viral RNA tissue loads, and clinical outcome in animals infected with either rNiV-B-ZsG or rNiV-B-BREP were not significantly different from those seen in wt-NiV-B infected animals (Fig. 6A–D, Supplemental Fig. 3). Our results corroborate reports of retained wild-type lethality following intraperitoneal infection with recombinant mCherry reporter NiV-M virus using a similar reporter expression strategy (Park et al., 2016). Future live imaging studies using rNiV-B-BREP and rNiV-M-BREP in small animal models can build and potentially improve upon the firefly luciferase-based reporter NiV-M imaging model (Escaffre et al., 2018; Yun et al., 2015).

In summary, we have successfully generated ZsG and BREP reporter NiVs and demonstrate their utility in antiviral screens by identifying and characterizing *in vitro* anti-NiV activity of OTA-4 in multiple cell lines. Furthermore, we show that these reporter viruses retain their wild-type pathogenicity in the Syrian hamster model, making them useful not only for evaluating potential therapeutics *in vivo*, but also for studying virus replication dynamics and pathogenesis in future *in vivo* and/or *ex vivo* imaging studies.

## Funding

This work was supported in part by CDC core Emerging Infections funding, by the Bill and Melinda Gates Foundation “Pathogenic Paramyxovirus Replication at BSL-4,” and by appointments to the CDC administered by the Oak Ridge Institute for Science and Education (ORISE) through an interagency agreement between the U.S. Department of Energy (DOE) and the USDA-ARS (K.A.D.). ORISE is managed by Oak Ridge Associated Universities (ORAU) under contract with DOE.

## Disclaimer

The content of this publication does not necessarily reflect the views or policies of the Department of Health and Human Services, nor does mention of trade names, commercial products, or organizations imply endorsement by the U.S. Government. The findings and conclusions in this report are those of the authors and do not necessarily represent the official position of the Centers for Disease Control and Prevention.

## CRediT authorship contribution statement

**Michael K. Lo:** Writing – review & editing, Writing – original draft, Visualization, Validation, Supervision, Resources, Project administration, Methodology, Investigation, Funding acquisition, Data curation, Conceptualization. **Shilpi Jain:** Investigation. **Katherine A. Davies:** Visualization, Investigation, Data curation. **Teresa E. Sorvillo:** Investigation. **Stephen R. Welch:** Investigation, Data curation. **JoAnn D. Coleman-McCray:** Investigation. **Payel Chatterjee:** Investigation. **Anne L. Hotard:** Investigation. **Troy O'Neal:** Investigation. **Mike Flint:** Resources. **Huiwang Ai:** Resources, Methodology. **Cesar G. Albariño:** Supervision, Resources, Methodology, Investigation. **Jessica R. Spengler:** Writing – review & editing, Writing – original draft, Supervision, Resources, Project administration, Methodology, Investigation, Funding acquisition, Conceptualization. **Joel M. Montgomery:** Writing – review & editing, Supervision, Funding acquisition. **Christina F. Spiropoulou:** Writing – review & editing, Supervision.

## Declaration of competing interest

The authors declare that they have no known competing financial interests or personal relationships that could have appeared to influence the work reported in this paper.

## Data Availability

Data will be made available on request.
