# Supplementary figures and images for: Optimization of Bangladesh and Malaysian genotype recombinant reporter Nipah viruses for in vitro antiviral screening and in vivo disease modeling
Source: Antiviral Res. 2024 Nov;231:106013. doi: 10.1016/j.antiviral.2024.106013 (PMC11772256; doi:10.1016/j.antiviral.2024.106013)

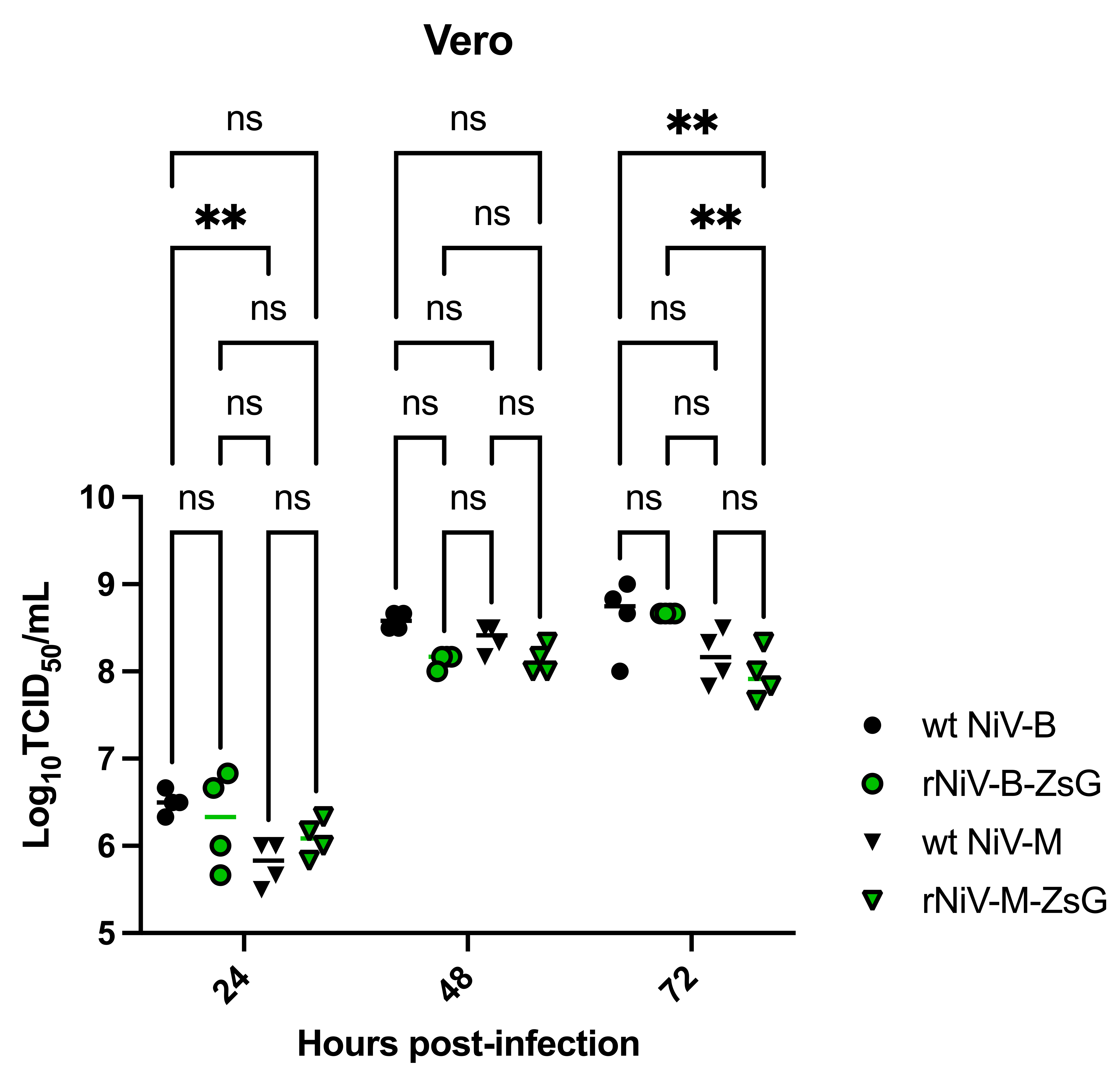

Supplement: Multimedia component 1 [file mmc1.zip › Supplemental Figures/Supplemental Figure 1 Vero Growth Curve Comparisons Individual Data.tiff]

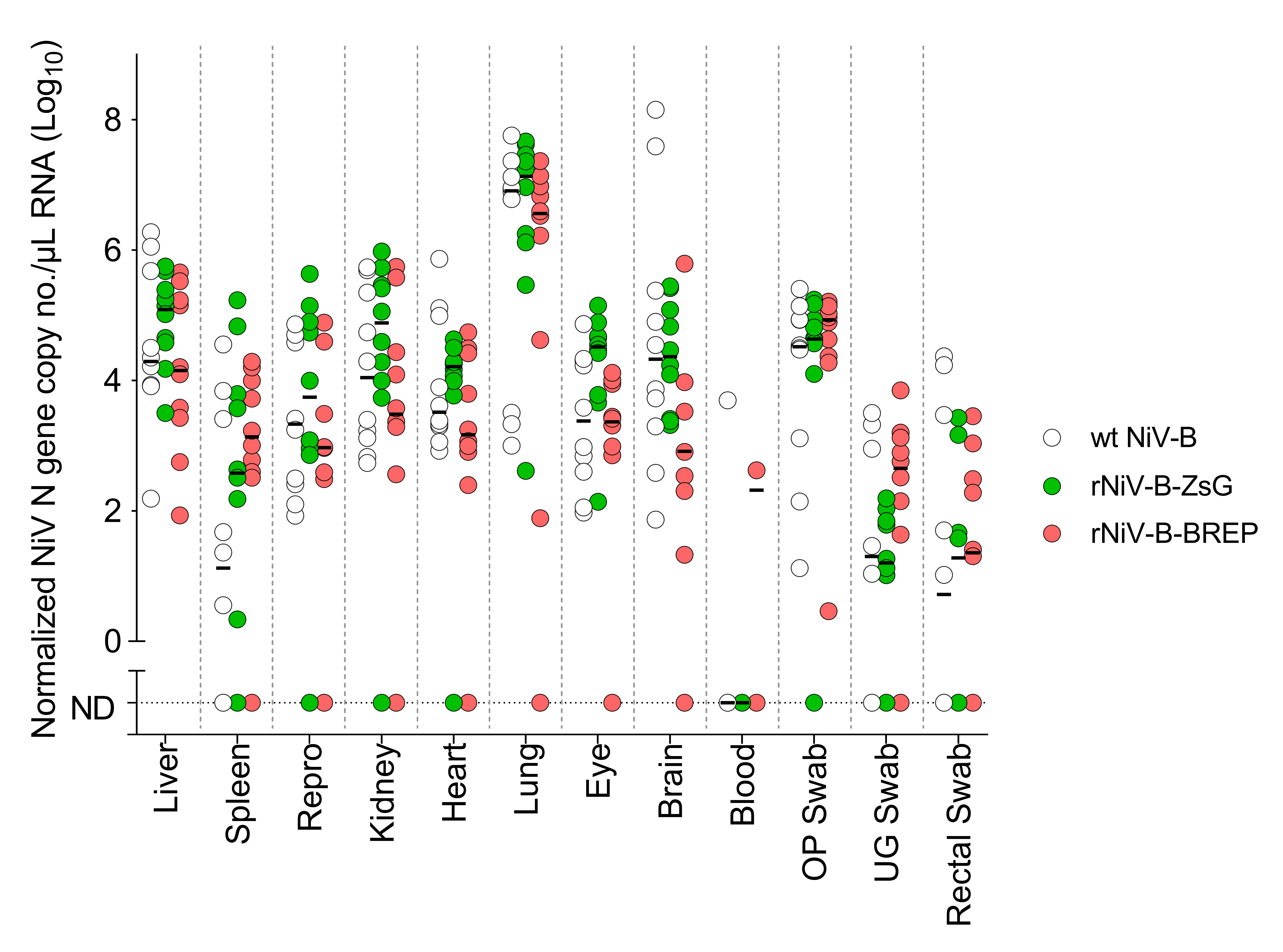

Supplement: Multimedia component 1 [file mmc1.zip › Supplemental Figures/Supplemental Figure 3 Tissue RNA.tif]
